# Older adults do not consistently overestimate their action opportunities across different settings

**DOI:** 10.1038/s41598-025-86790-6

**Published:** 2025-02-07

**Authors:** Isabel Bauer, Milena S. Gölz, Lisa Finkel, Maddalena Blasizzo, Sarah E. M. Stoll, Jennifer Randerath

**Affiliations:** 1 Department of Psychology, University of Konstanz, Constance, Germany; 2 Lurija Institute for Rehabilitation Science and Health Research, Kliniken Schmieder, Allensbach, Germany; 3 Psychotherapy Training Center Bodensee (apb), Konstanz, Germany; 4 Department of Developmental and Educational Psychology, Faculty of Psychology, University of Vienna, Vienna, Austria; 5 Clinical Neuropsychology & Neuropsychological Psychotherapy, Institute of Psychology, University of Regensburg, Regensburg, Germany

**Keywords:** Affordance judgments, Aging, Judgment tendency, Overestimation, Underestimation, Psychology, Human behaviour

## Abstract

Am I still able to climb the ladder? Aging accompanies changes in physical constitution and a higher risk of injuries. At the same time, the judgment of action opportunities needs to be highly adaptive to the given task setting. We examined older adults’ (*n* = 40) judgment tendencies in four different tasks by use of a detection theory approach. The tasks’ setting differed in their boundaries’ proximity to the actor with either proximal (e.g., judging one’s hand fit into an opening) or distal boundaries (e.g., judging the reachability of a distant object). The older participants showed significantly more liberal judgments in tasks with distal boundaries. Body awareness and alertness were associated with the extent of judgment disparity between setting types. Subsequently, we compared a gender- and education-matched subsample of the group (*n* = 24) to a younger sample (*n* = 24). Older participants’ judgment tendencies were significantly more extreme, with stronger under- or overestimations depending on the type of setting. We discuss potential links between more extreme judgments in older adults and higher reliance on learned patterns. Future research is needed to further unravel these setting-dependent behavioral differences and the factors contributing to more extreme judgment tendencies with growing age.

**Supplementary Information:**

The online version contains supplementary material available at 10.1038/s41598-025-86790-6.

## Introduction

Aging is associated with a higher risk of falls and injuries^1,2^. On the one hand, bodily vulnerability is higher in older adults due to bodily changes with age, such as declining bone density^3^ or reduced muscle mass^4^. On the other hand, declines in mobility, strength, or balance reduce safe actions^5,6^. Both aspects result in some actions being increasingly more dangerous for older adults, such as climbing a ladder or overcoming obstacles. For example, George Bernard Shaw, a 94-year-old Irish writer who still engaged in active gardening, died following a fall from a ladder. Beyond such prominent cases, everyone might know older people who seem to overestimate their capabilities and stick to activities that the social environment considers rather dangerous with regard to their bodily conditions, while other older persons seem overly cautious. This raises the question of how older adults judge their action opportunities in different settings. To safely judge what actions the environment and one’s own capabilities afford, one needs to take environmental properties into account and estimate the capabilities and constraints adequately^7^. A healthy young person might perceive stairs as climbable, whereas a person with walking difficulties may not perceive them as climbable, and a misjudgment could lead to injuries. While studies show that healthy young adults make fairly accurate judgments on their action opportunities in different tasks^8,9^, e.g., when deciding whether a narrow opening can be passed, a different picture emerges for older adults. Several studies applying different tasks report the tendency of older adults to overestimate their capabilities of reaching^10^, stepping over obstacles^11–13^, balance^14^, walking ability^15–17^, or crossing a street^18^. The misjudgment of stepping-over ability, stepping-forward ability and walking speed is, in turn, associated with risk of falls^11,19,20^.

Research on the adaptation of adults’ judgments to changing sensorimotor contexts shows that young, healthy adults are capable of quickly adapting their judgments on changing body properties, for example, when being equipped with a hand splint^21^. In this study, healthy younger and healthy older participants had to judge whether their hand might fit into a narrow opening, either with or without a splinted hand. As compared to younger adults, older adults seemed to have more difficulties by showing less accurate judgments right after the change in body properties^21^. A recent study by Luyat et al. examined the after-effects of tool use in younger and older adults^10^. This ‘tool effect’ of an overestimation in a reaching task after using a tool was shown for younger adults and seems to reflect the perceptual-motor system’s high plasticity^10,22^. However, a higher overestimation after using a tool was absent in older adults^10^. The authors suggest that the frequently reported overestimation of motor skills in older adults might result from reduced adaptability of the perceptual-motor system^10^.

However, not all tasks seem to be associated with an overestimation of action opportunities in older adults. For example, older adults showed a rather conservative judgment tendency (underestimation) in an aperture task, in which participants decided whether their hand would fit into openings with varying widths^21,23^. While perceptual sensitivity, the ability to discriminate between a fit and a non-fit, was similar to younger adults in these studies, judgment tendencies were more conservative, reflecting more cautious judgments. In another study, especially older women showed rather conservative judgments when deciding whether to walk through openings with varying size^24^. Similarly, Hackney and Cinelli reported a rather cautious behavior of older adults when walking through doorways^25^. These differing results in different affordance judgment tasks may indicate that there is no general age specific judgment tendency (over- or underestimation) in affordance judgments, but rather a significant effect by task type. Kluft et al. investigated the consistency of misjudgment in older adults in four different stepping-tasks^26^, which according to Finkel et al. would be categorized as tasks with distal boundaries^9^. The authors reported no transfer of misjudgment behavior across these homogenous tasks and suggested that misjudgments might be task-dependent instead. Thus far, there is a lack of studies that specifically look at differences in judgment tendency in older adults across motor tasks with different task-related boundaries.

As a result of their literature review on actor-related affordances, Finkel et al. proposed that different affordance judgment tasks may differ in the primary processing of boundaries that can be either proximal-directed or distal-directed^9^. For proximal-directed tasks, the affordance judgments are predominantly guided by individual body dimensions. For example, hand size is crucial for the decision of whether the hand can be put through an opening and body height limits the maximum height of a ceiling a person can stand upright under. In fitting tasks, such as passing through doorways or fitting a body part in a narrow opening, the task-related boundaries are directed towards the body, which the review by Finkel et al. shows to be predominantly associated with underestimation of abilities^9^. In contrast, for distal-directed tasks, environmental boundaries predominantly guide affordance judgments. This applies to tasks in which a person needs to decide for example upon the maximum distance of an object that can be reached or the maximum height of a hurdle that can be crossed. Thus, for some tasks, the boundaries that limit the action are rather directed away from the body with a focus on overcoming environmental challenges^9^. As examples, the authors mention crossing obstacles or reaching for objects, which are associated with an overestimation of abilities (liberal judgment tendency). Also, from an evolutionary perspective, the distinction between proximal and distal task-related boundaries is plausible. It seems beneficial to be rather courageous when it comes to overcoming obstacles, potentially expanding ranges, or trying to reach, e.g., a fruit from a tree. Pushing oneself to the limits by tending to overestimate one’s own abilities may also have a training aspect, potentially increasing capabilities for future challenging situations. In contrast, in situations with proximal task boundaries that are directed towards the body, it seems more beneficial to show a rather cautious behavior in order to, e.g., prevent getting stuck. The negative consequences of getting stuck appear clearly higher than being unsuccessful while reaching or surpassing an object, at least as long as sufficient stabilizing capabilities prevent us from falling.

The current study addresses the question of how older persons tackle different types of affordance judgment tasks that have been categorized into actions with rather proximal versus distal boundaries^9^. Previous studies used different designs and approaches to quantify judgment tendencies. Therefore, a controlled within-subject design including different task types and the same approach of measuring judgment tendencies was required to enable a more conclusive evaluation of judgment behavior across different tasks^9^. With the current work, we aimed to fill the gap by examining a sample of older persons in four different affordance judgment tasks. The study focuses on analyzing judgment tendencies (criterion, c) as a measure of potential bias to assess whether participants show a rather conservative (underestimation) or a rather liberal (overestimation) judgment behavior^27,28^. Further, we describe judgment performance by use of the variables accuracy (% accurate judgments) and perceptual sensitivity (d-prime, d’)^27,28^. The latter quantifies the ability to discriminate between possible and impossible actions. Two of the applied tasks (Aperture Task, Fit Under Task) are characterized by proximal boundaries, and two of the applied tasks (Hurdle Task, Reach Task) are characterized by distal boundaries^9^.

First, based on the literature^9^, we hypothesized that older participants show a significantly more conservative judgment tendency in tasks with proximal boundaries (Aperture Task, Fit Under Task) than in tasks with distal boundaries (Hurdle Task, Reach Task). To explore potentially influential cognitive factors, we checked for associations between judgment tendencies in the affordance judgment tasks and general risk-perception and risk-taking, body awareness, and performance in a computerized neuropsychological test battery.

In a second step, we examined the specific role of age in over- and underestimations in the four applied tasks. To do so, we compared a subgroup of our sample of older participants to a gender- and education-matched control sample of younger adults, who had participated in a previously conducted study. Thus far, several studies comparing older participants with younger adults showed rather conservative judgments in tasks with proximal boundaries in older participants^23–25^, but more liberal judgments in tasks with distal boundaries^10–12^. We expected to find similar task-related group differences for our within-subject design implementing the four distinct tasks: the older participants were expected to show a more conservative judgment tendency in our tasks with proximal boundaries (Aperture, Fit Under), and a more liberal judgment tendency in our tasks with distal boundaries (Hurdle, Reach).

## Methods

### Participants

All participants gave informed written consent before voluntarily taking part in this study and received financial compensation for their participation. Older participants were mainly invited via an advertisement in the local newspaper (Südkurier) as well as via notices in, e.g., local senior citizens centers and ecclesiastical institutions. Younger subjects were invited via Sona Systems (https://www.sona-systems.com) and by announcement in the university building. Data collection of the older sample took place between January 10, 2022 and August 30, 2022. Data from the younger sample was collected between June 13, 2019 and June 22, 2021.

Participants reported no history of neurologic or psychiatric disorders, except for two participants who reported depressive symptoms in the past with no current symptoms or medication. All participants were right-handed (lateralization quotient^29^) and had normal or corrected-to-normal vision. Due to setting requirements, only participants with a body height of a minimum of 150 cm and a maximum of 185 cm were invited. By use of the DemTect^30^ moderate to severe cognitive impairment was ruled out (total scores ≥ 11). In sum, data sets of 40 older participants (20 female) between 61 and 81 years of age *M* = 69.50 years (*SD* = 5.02) were analyzed.

To compare older participants’ performance to a younger sample, we retrieved data from a previous study in our lab^31^. 24 healthy younger participants (12 female) between 18 and 32 years of age (*M* = 22.92, *SD* = 3.37) served as education- and gender-matched controls. As all younger participants had a high school diploma (Abitur) or were higher educated, older participants with less education than a high school diploma were excluded from the between-subject analysis between the younger and the older sample. Consequently, all participants of the matching groups had at least a high school diploma. The resulting subsample of *n* = 24 older adults (12 female) was aged between 61 and 81 years (*M* = 68.75, *SD* = 4.72).

### General procedure and material

The project was approved by the Ethics Committee of the University of Konstanz (#15/2020) and conducted in accordance with the Declaration of Helsinki. The main tasks (four affordance judgment tasks: Aperture Task, Fit Under Task, Hurdle Task, Reach Task) were administered in one session of approximately 120 min. In a second session, subjects accomplished a neuropsychological test battery of approximately 120 min. Each older participant accomplished both sessions within a maximum of two weeks.

**Fig. 1:**
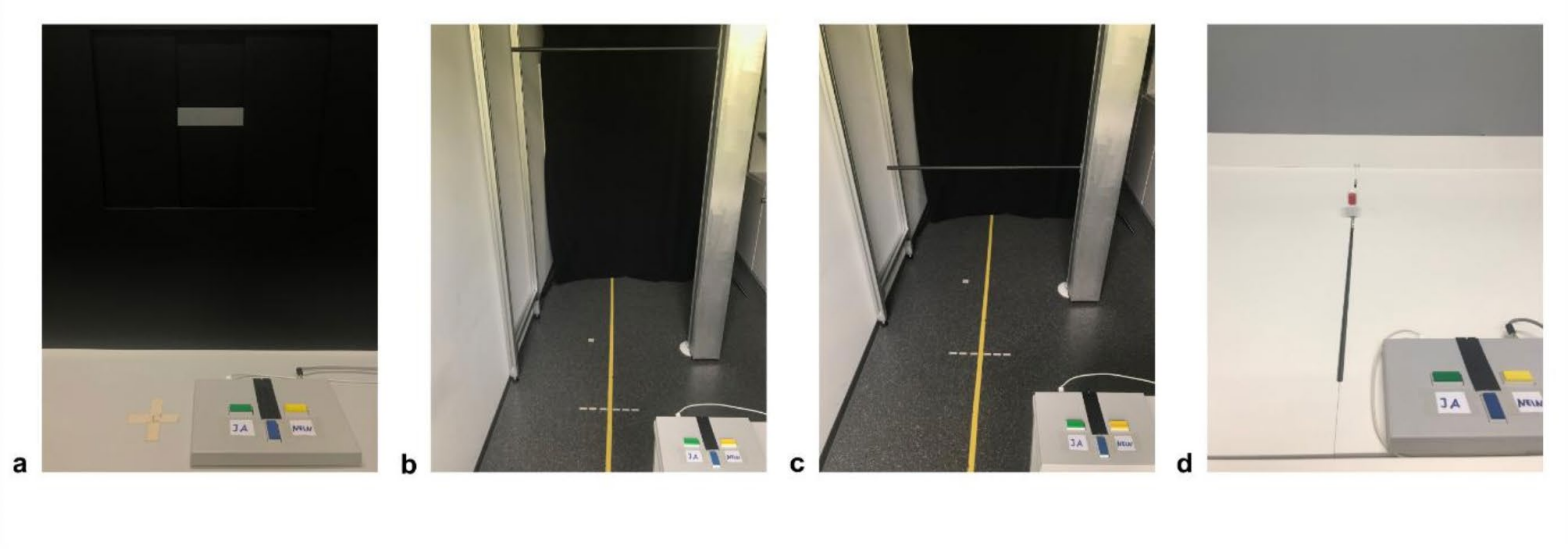
Experimental setting of the Aperture Task (**a**), Fit Under Task (**b**), Hurdle Task (**c**) and Reach Task (**d**).

The experimental material of the four affordance judgment tasks was custom-made. Material and measurement procedure of the Aperture Task and the Reach Task were adapted from previous studies^21,32,33^. The design of the Fit Under Task was similar to Marcilly and Luyat^34^. The design of the Hurdle Task was similar to Petrucci et al.^35^. Across all four tasks, participants gave their response (whether they thought they would be able to perform the respective action or not) via a response pad providing participants two response options (Cedrus, RB540; see Fig. 1). A green button marked “Yes” was pressed for actions estimated as possible and a yellow button marked “No” for actions estimated as not possible. Judgments were given using the right hand. During the affordance judgment tasks, participants wore Plato goggles (Translucent Technologies Inc.) to control vision. To prevent visual feedback, goggles were switched to opaque during the measurement of the actual individual reference (0-trial setting) in the beginning of each of the four main tasks (Aperture Task, Fit Under Task, Hurdle Task, Reach Task) as well as between trials for the time a new adjustment of the setup was made. Trial protocol-related adjustments were either programmed and regulated by a computer-controlled motor (Aperture, Reach) or set manually (Fit Under, Hurdle). SuperLab 5 Software (provided by Cedrus) was used for coding experimental data.

Each of the four tasks started out with the measurement of the actual individual reference, i.e., hand size (Aperture Task), body height (Fit Under Task), maximum step-over ability (Hurdle Task) or maximum reachability distance (Reach Task). This was followed by an introductory set of 20 trials, as earlier studies indicated that a rather stable judgment tendency is established in a familiarization phase^32^. Afterward, 30 experimental trials were completed. Note that only the experimental trials were included in the data analysis. Participants were instructed to make their judgments as precisely as possible. While participants performed the Fit Under, Hurdle and Reach Task standing, they sat on a chair during the Aperture Task. Participants received no feedback at any time during the four affordance judgment tasks on whether the respective action to be judged actually was possible or not.

### Tasks and procedure

#### Aperture Task

In the Aperture Task, participants had to decide whether they would be able to put their flat hand through a presented opening. Thus, participants’ individual hand size served as reference for the task and was measured in the beginning. The aperture apparatus was built with a rectangular opening in the center that could be adjusted in horizontal and vertical size to adjust for individual hand size. The experimental setup is depicted in Fig. 1a. Participants were instructed to judge as precisely as possible whether they would be able to put their hand through the presented aperture with varying horizontal size. The aperture either was large enough to fit the hand by representing the actual hand size or wider openings (increments: ±0, + 2, +4, + 8, +16 mm) or did not enable the action by representing smaller openings than the actual hand size (increments: -2, -4, -8, -16 mm). The trials with an increment of 0 showed the minimum horizontal width the hand fitted through (thus the participant’s measured hand width). To hold the number of correct yes-answers (fit) and correct no-answers (non-fit) constant, filler trials were included (increment: -30 mm). These more extreme trials were excluded from further analysis. The vertical size of the aperture was set to the participants’ actual vertical hand size.

#### Fit Under Task

In the Fit Under Task, participants had to decide whether they could stand upright under a horizontal rod. Thus, the participants’ body height served as a reference for the task and was measured in the beginning. The device for the Fit Under Task consisted of a mount enabling the adjustment of an aluminum rod horizontally to differing heights precise to the millimeter. This was facilitated by a metal rail that allowed easy sliding up and down along a scale. A fine indicator pointing to the scale together with a screw handle allowed the tightening at the correct position. Behind the device with the aluminum rod, a black curtain prevented visual reference. The experimental setup is depicted in Fig. 1b. The aluminum rod measured one meter in length and was equipped with an oval boundary surface facilitating body height measurement. Participants had a distance of 150 cm from the rod. The rod’s height either enabled the action by representing the actual body height or higher heights (increments: ±0, + 5, +10, + 20, +40 mm) or did not enable the action by representing lower heights than the actual body height (increments: -5, -10, -20, -40 mm; extreme filler trials: -80 mm).

#### Hurdle Task

In the Hurdle Task, participants had to decide whether they would be able to step sideways over a horizontal rod without jumping. Thus, participants’ maximum step-over height served as a reference and was measured in the beginning. None of the older participants were able to lift their foot sideways higher than their crotch height. Therefore, in the older sample, the maximum step-over height was determined by the maximal lifting height of the foot. In contrast, all of the younger participants were able to lift their foot higher than their crotch height. As the step-over height is in this case limited by the crotch height, we measured the crotch height as most appropriate measure for maximal step-over height in the younger sample. For the Hurdle Task, the same device as for the Fit Under Task was used. Only the used aluminum rod of one meter length was not equipped with an oval boundary surface. The experimental setup is depicted in Fig. 1c. Similar to the Fit Under Task, participants had a distance of 150 cm from the rod. The rod’s height either enabled the action by representing the actual maximum step-over height or lower heights (± 0, -20, -40, -80, -160 mm) or did not enable the action by representing higher heights than the actual maximum step-over height (+ 20, + 40, +80, + 160 mm; extreme filler trials: +300 mm).

#### Reach Task

In the Reach Task, participants had to decide whether they would be able to reach an object in front of them by stretching their arm forward. Thus, participants’ maximum reachability distance served as a reference and was measured in the beginning with the help of a measurement tape mounted on the table. The reach apparatus was built on a height-adjustable table. Three tracks were mounted onto it with one rectangular object on each track. For the current study, only the middle track was used. The objects could be presented at varying distances and were automatically controlled by a motor. A spacer at the front end of the table allowed to account for different arm lengths. The experimental setup is depicted in Fig. 1d. Participants stood right in front of the reach apparatus. The height of the apparatus prevented participants from leaning forward. The distance of the object either enabled the action by representing the participants’ actual maximum reachability distance or more proximate distances (± 0, -20, -40, -80, -160 mm) or did non enable the action by representing more remote distances (+ 20, + 40, +80, + 160 mm; extreme filler trials: +300 mm).

The sequence of the applied tasks was balanced, and participants were randomly assigned to different sequences of the tasks.

#### Control tasks

##### Gradual body-related size estimation task

Participants’ size estimation of the respective body parts relevant to the affordance judgment task might be associated with their performance in the task. Therefore, we administered a gradual size estimation task after each of the four main tasks. In the Aperture Task, the horizontal size of the opening was either gradually decreased (starting at 0 mm) or increased (starting at 200 mm), two trials each. Participants verbally indicated to stop the gradual closing or opening of the aperture as soon as they estimated the horizontal width to exactly reflect their hand’s widest part. They were allowed to check the opening again and demand to open or close the opening further until they were satisfied with the set horizontal size. In the Fit Under Task, the horizontal rod was gradually put up or down until participants estimated it to be at their body height. In the Hurdle Task, the horizontal rod needed to be adjusted to the upper end of the pelvic bone. In the Reach Task, participants adjusted a measuring tape until it reflected their estimated arm length.

##### Gradual object-related estimation task

For each of the four affordance judgment tasks, another control task was administered that required an estimation judgment with respect to an object instead of participants’ own body to examine object-related perception and account for a potential visual perception bias. As for the body-related control tasks, settings (of the opening/ height/ distance) were approached gradually to the target position until participants indicated to stop. For the Aperture Task, participants performed the object-related estimation task for a small wooden cuboid (105 mm x 105 mm x 30 mm). They were instructed to say stop as soon as they estimated the gradual opening or closing of the aperture (starting either from 0 mm or from 200 mm) to match the size of the cuboid. For the Fit Under Task, the height of a wooden block (800 mm x 200 mm x 200 mm) needed to be indicated from a sitting position. Participants were instructed to say stop as soon as they estimated the gradually ascending or descending horizontal rod to be at the same height as the wooden block. For the Hurdle Task, the height of the same wooden block was supposed to be estimated from a standing position. For the Reach Task, participants aligned the position of the presented movable object on the track to a fixed object that was put on the right side next to the track with a distance of 495 mm from the back end of the reach apparatus. The starting position of the movable object on the track was either closer to the participant or farther away from the participant than the fixed object.

#### Additional assessments

##### Neuropsychological test battery

To exploratively check for associations between affordance judgment behavior in the applied tasks and cognitive variables, we assessed a computerized neuropsychological test battery out of the Vienna Test System (VTS, Version 8)^36^ that included the following tests: 3D (spatial orientation)^37^, CORSI (corsi block-tapping test backwards)^38^, FGT (figural memory test)^39^, LAT (line orientation test)^40^, NBV (N-back verbal)^41^, SIGNAL (signal detection)^42^, TOL-F (tower of London – Freiburg version)^43^, VISCO (visuoconstruction test)^44^, WAF Alertness (perception and attention function battery)^45^, WIWO (Vienna verbal fluency test)^46^.

##### Body awareness

We assessed participants’ body awareness by use of the German version of the Body Awareness Questionnaire^47,48^. The questionnaire consists of 18 items assessing self-reported sensitivity to normal body processes (e.g. “I can always tell when I bump myself whether or not it will become a bruise.”, “I always know when I’ve exerted myself to the point where I’ll be sore the next day.”).

### Data analysis

Behavioral data were analyzed with SPSS 28 (IBM).

#### Dependent variables measuring affordance judgment behavior

We analyzed the detection theory^27,28^ parameter judgment tendency (criterion c) as a measure of potential response bias, indicating whether participants applied a rather conservative (positive values of c) or a rather liberal (negative values of c) judgment tendency. A conservative judgment tendency is reflected by positive criterion values (more “no” responses in comparison to an ideal observer; underestimation of one’s ability). A liberal judgment tendency is reflected by negative criterion values (more “yes” responses in comparison to an ideal observer; overestimation of one’s ability). In order to describe participants’ performance beyond judgment tendency, we additionally analyzed general accuracy (percent correct) and perceptual sensitivity (d’), another signal detection theory parameter, which served as a measure to determine participants’ ability to discriminate between a fit (possible action) and a non-fit (impossible action). Judgment tendency and perceptual sensitivity are two independent parameters and were calculated from the following formulas: c = -0.5 × [*z*(Hit rate) + (*z*(False-Alarm rate)]; d’ = *z*(Hit rate) – *z*(False-Alarm rate)^27,28^.

Shapiro-Wilk test results as well as screening of normal probability plots indicated that normal distribution could not be assumed (see Supplementary Table S1 online for Shapiro-Wilk test results). As a consequence, data were analyzed by use of non-parametric procedures. For Friedman test results, asymptotic p-values are reported. For all further test results, exact p-values are reported. For analyses based on our specific hypotheses, one-tailed p-values are reported. For all other analyses, p-values are reported two-tailed. G*Power^49^ was used to compute statistical power (1-β; as the complement of Type II error magnitude). Test power was calculated with an alpha level of 0.05, two-tailed for accuracy and perceptual sensitivity and, corresponding to our specific hypotheses, one-tailed for judgment tendency.

#### Within subject analyses

In order to provide a general description of older participants’ judgment performance, we firstly report general accuracy results (percent correct) as well as perceptual sensitivity results (d’, ability to discriminate between a possible and an impossible action) and subsequently report results referring to our specific hypotheses on judgment tendency (c). We ran Friedman tests to evaluate whether there was a main effect of type of task (Aperture, Fit Under, Hurdle, Reach) per variable (accuracy, perceptual sensitivity, judgment tendency). For significant results, we ran post-hoc Wilcoxon signed ranks tests to further specify differences between each pair of tasks. For statistical analyses of judgment tendency between tasks with proximal (Aperture, Fit Under) versus distal (Hurdle, Reach) boundaries, we ran one-sided tests corresponding to our specific hypotheses. The remaining comparisons were performed two-sided. The stepwise Holm-Bonferroni procedure was applied to correct for family-wise error rate. Effect size *r* was calculated by use of Wilcoxon *z*-values by dividing *z* by the square root of *N*, as proposed by Cohen^50^. Note that *N* corresponds to the number of observations^51^ (*N* = 80).

#### Correlations

In order to explore potential factors that might influence older participants’ judgment tendencies or extremity of judgment tendencies between tasks, we post-hoc explored correlations between judgment tendencies and results of size estimation, cognitive variables, and body awareness. As will be described in the results, against expectations, participants showed a rather liberal judgment tendency in the Fit Under Task. The divergent role of the Fit Under Task is also reflected in the significant difference in older participants’ judgment tendency in the Fit Under Task as compared to the Aperture Task, i.e. the two tasks with proximal boundaries. We consequently excluded the Fit Under Task from post-hoc correlational analyses on the extremity of judgment tendencies in relation to body awareness scores and cognitive variables.

##### Size estimation

To test for a correlation between judgment tendency and size estimation of one’s own body, respectively object size estimation in the affordance judgment tasks, Kendall’s tau was calculated between judgment tendency (c) and gradual size estimation tasks (control tasks) for each task (Aperture, Fit Under, Hurdle, Reach).

##### Cognitive variables

Two participants did not complete the entire test set due to slow progression. For one participant, one test was missing and for another participant, two tests were missing (see Supplementary Table S2 online). To reveal potential cognitive variables associated with extremity of judgment tendency, we post-hoc explored correlations (Kendall’s tau) between deltas of judgment tendencies (difference scores) between tasks with proximal vs. distal boundaries (e.g., Aperture-Hurdle) and performance in the applied tests of the neuropsychological test battery of the VTS^36^ (see section “neuropsychological test battery”) above.

##### Body awareness scale

One older participant did not complete the body awareness questionnaire and therefore was not included in the analysis on body awareness. We calculated Kendall’s tau between body awareness scores^47,48^ and deltas of judgment tendencies between tasks (e.g. Aperture-Hurdle).

#### Additional between-subject analyses – older vs. younger sample

To compare older adults’ judgment tendency in the four applied tasks with a younger sample, we ran Mann-Whitney tests for each task between the older (*n* = 24) and a younger (*n* = 24) sample. Corresponding to our specific hypotheses, we ran one-sided Mann-Whitney *U*-tests for judgment tendency. Accuracy and perceptual sensitivity were analyzed by two-sided Mann-Whitney *U*-tests. Note that we compared the younger sample only to a subsample of the older participants to ensure matching with regard to gender and education (see section “Participants”). Descriptive statistics of affordance judgment performance variables were similar between the whole sample (*n* = 40) and the subsample (*n* = 24) of older participants (cf. Tables 1 and 4). With regard to judgment tendency, there was only a slight tendency of more liberal judgments across tasks in the subsample. Similar to within-subject analyses, effect size *r* was calculated by dividing *z*-values by the square root of *N* (*N* = 48)^50,51^.

**Table 1:** Descriptive data for older participants (*n* = 40) per task and variable.

Variable	Aperture	Fit Under	Hurdle	Reach
	*M*_*dn*_ *[IQR]*	*M*_*dn*_ *[IQR]*	*M*_*dn*_ *[IQR]*	*M*_*dn*_ * [IQR]*
acc	77.78 [71.30, 85.19]	74.07 [62.96, 80.56]	70.37 [55.56, 81.48]	64.81 [59.26, 77.78]
d’	1.84 [1.57, 2.35]	1.48 [0.93, 1.88]	1.44 [0.13, 2.08]	1.07 [0.48, 1.86]
c	0.53 [-0.32, 0.84]	-0.84 [-1.28, -0.31]	-1.16 [-1.82, -0.84]	-1.28 [-1.64, -0.76]

acc = accuracy, d’ = perceptual sensitivity, c = judgment tendency, *M*_*dn*_ = median,

*IQR* = interquartile range.

##### Domain-specific risk-taking

We applied the health and safety subdomain of the domain-specific risk-tasking scale (DOSPERT)^52,53^, as this scale has been shown to be associated with affordance judgment behavior with regard to age differences^23^. The health and safety subdomain consists of three parts, six items each (e.g., “Riding a motorcycle without a helmet”, “Drinking heavily at a social function”). In the different parts, participants rated on a 7-point Likert Scale the likelihood of engaging in risky activities (risk-taking; highly unlikely to highly likely), the magnitude of these risks (risk-perception; absolutely no risk to very high risk), and the benefit of engaging in these risks (no benefit at all to high benefit). We only analyzed data for the risk-taking subscale and the risk-perception subscale. One younger participant did not complete the risk-perception subscale and therefore was not included in the analysis on risk-perception. We compared DOSPERT questionnaire scores for risk-taking and risk-perception of the health and safety subdomain between the older and the younger sample by use of one-sided Mann-Whitney *U*-tests.

## Results

### Performance variables

For all three main variables, Friedman tests revealed a main effect of task (accuracy: χ^2^(3) = 12.54, *p* = .006; perceptual sensitivity: χ^2^(3) = 20.32, *p* < .001, judgment tendency: χ^2^(3) = 63.96, *p* < .001).

**Fig. 2:**
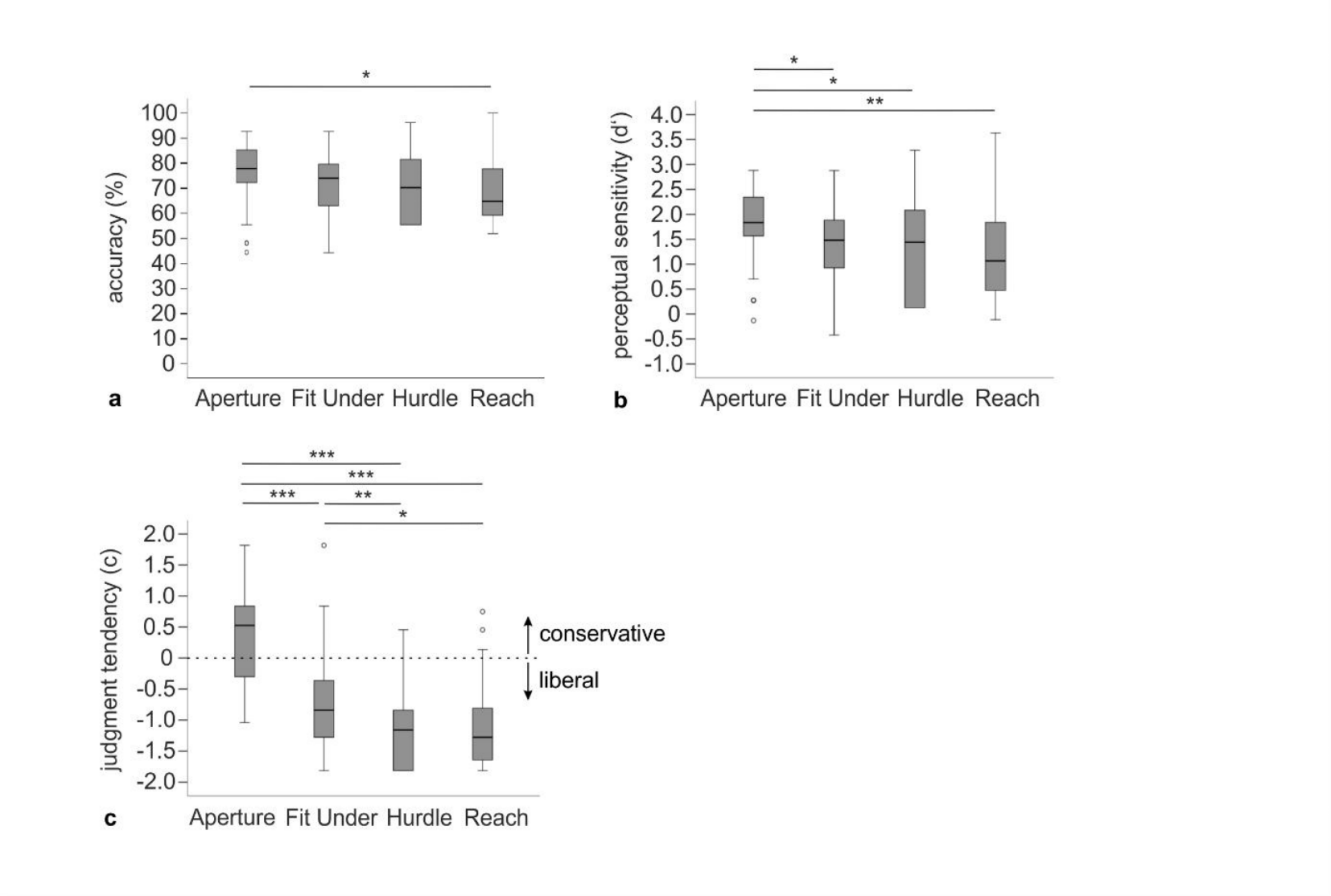
Boxplots for performance variables in older participants (*n* = 40). (**a**). Boxplots for accuracy (percent correct) per task. (**b**). Boxplots for perceptual sensitivity (d’) per task. Higher values stand for better perceptual sensitivity. (**c**). Boxplots for judgment tendency (**c**) per task. Values closer to zero reflect a more ideal judgment tendency. Positive values stand for a rather conservative judgment tendency and negative values for a rather liberal judgment tendency. Note that *p*-values are reported one-tailed for judgment tendency, corresponding to our specific hypotheses. *Note.* * *p* < .05, ** *p* < .01, *** *p* < .001 (after Holm-Bonferroni adjustment).

For accuracy, Wilcoxon signed rank tests showed a significant difference between the Aperture and the Reach Task. After Holm-Bonferroni adjustment, there was no significant difference between the other tasks. See Table 1 for descriptive data, Table 2 for detailed statistics and Fig. 2a for boxplots.

**Table 2:** Post-hoc Wilcoxon signed ranks test results for task differences in accuracy, perceptual sensitivity and judgment tendency between the four applied tasks.

Tasks	Variable	Fit Under					Hurdle					Reach				
		*z*	*p*	*p*_***adj***_	*1- β*	*r*	*z*	*p*	*p*_***adj***_	*1- β*	*r*	*z*	*p*	*p*_***adj***_	*1- β*	*r*
Aperture	acc	1.89	0.058	0.232	0.965	0.21	2.35	0.017	0.087	0.998	0.26	2.67	0.007	0.040	< 0.999	0.30
	d’	2.76	0.005	0.020	< 0.999	0.31	3.02	0.002	0.010	< 0.999	0.34	3.20	< 0.001	0.006	< 0.999	0.36
	c	4.38	< 0.001	< 0.001	< 0.999	0.49	5.50	< 0.001	< 0.001	< 0.999	0.61	5.39	< 0.001	< 0.001	< 0.999	0.60
Fit Under	acc						0.57	0.578	0.578	0.192	0.06	1.01	0.319	0.956	0.500	0.11
	d’						0.74	0.468	0.468	0.293	0.08	1.17	0.247	0.740	0.619	0.13
	c						2.77	0.002	0.007	< 0.999	0.31	2.16	0.015	0.030	0.997	0.24
Hurdle	acc											0.94	0.352	0.703	0.444	0.11
	d’											0.84	0.406	0.812	0.367	0.09
	c											0.17	0.868	0.868	0.094	0.02

acc = accuracy, d’ = perceptual sensitivity, c = judgment tendency. Both *p*-values and Power (*1-β*) are reported two-tailed for acc and d’, and one-tailed for c. Power was calculated by using G*Power^49^. *p*_*adj*_ = *p*-values after Holm-Bonferroni adjustment.

For perceptual sensitivity, Wilcoxon signed ranks tests showed significant differences between the Aperture Task compared to the Fit Under, Hurdle, and Reach Task, with higher perceptual sensitivity in the Aperture Task (see Fig. 2b; Tables 1 and 2). There was no significant difference in perceptual sensitivity between the Fit Under Task and the Hurdle or the Reach Task.

Concerning judgment tendency, Wilcoxon signed rank tests showed a significantly higher criterion in the Aperture Task compared to the Fit Under, Hurdle, and Reach Task as well as in the Fit Under Task compared to the Hurdle and Reach Task (see Fig. 2c; Tables 1 and 2). There was no significant difference between the Hurdle and the Reach Task.

#### Control tasks

With regard to the Aperture Task and the Fit Under Task, there was a significant correlation between judgment tendency and body size-related estimation. A higher overestimation (or lower underestimation) of older participants’ own hand size was associated with more conservative judgments in the Aperture Task. In the Fit Under Task, the more the older participants underestimated their body size (or the less they overestimated), the more liberally they decided. In contrast, there was no significant correlation between judgment tendency and object size-related estimation. Also, no significant correlations were found for the Hurdle and Reach Task with their respective body-related or object-related gradual size estimation tasks. See Table 3 for detailed statistics.

**Table 3:** Correlations between control task (body-related, object-related) results and judgment tendency performance in the four main tasks.

	Aperture		Fit Under		Hurdle		Reach	
	τ_b_	*p*	τ_b_	*p*	τ_b_	*p*	τ_b_	*p*
Body-related control task	-0.31	0.006	-0.41	< 0.001	0.15	0.202	0.22	0.051
Object-related control task	0.01	0.944	-0.09	0.421	-0.10	0.382	0.04	0.707

*Note.* For each main task, a task-specific body-related and object-related control task was administered (see Methods section).

#### Correlations with neuropsychological tests

There was a significant correlation between Alertness (WAF) and difference scores for deltas including the Aperture Task (*n* = 40; Aperture-Hurdle: τ_b_ = 0.38, *p* = < 0.001; Aperture-Reach: τ_b_ = 0.24, *p* = .030). The lower the older participants’ alertness, the higher the delta of judgment tendencies between tasks. There was no significant correlation between difference scores and the other analyzed VTS tests (see Supplementary Table S2 online).

#### Correlations with body awareness

Older participants (*n* = 39) showed a significant correlation between body awareness scores and deltas of judgment tendencies between the Aperture Task and the Hurdle Task (τ_b_ = 0.30, *p* = .008). The higher participants’ body awareness, the higher the delta between the two tasks. For the delta between the Aperture and the Reach Task, there was only a tendency towards a higher delta of judgment tendencies for higher scores of body awareness on a descriptive level (τ_b_ = 0.22, *p* = .051).

#### Older versus younger sample

**Fig. 3:**
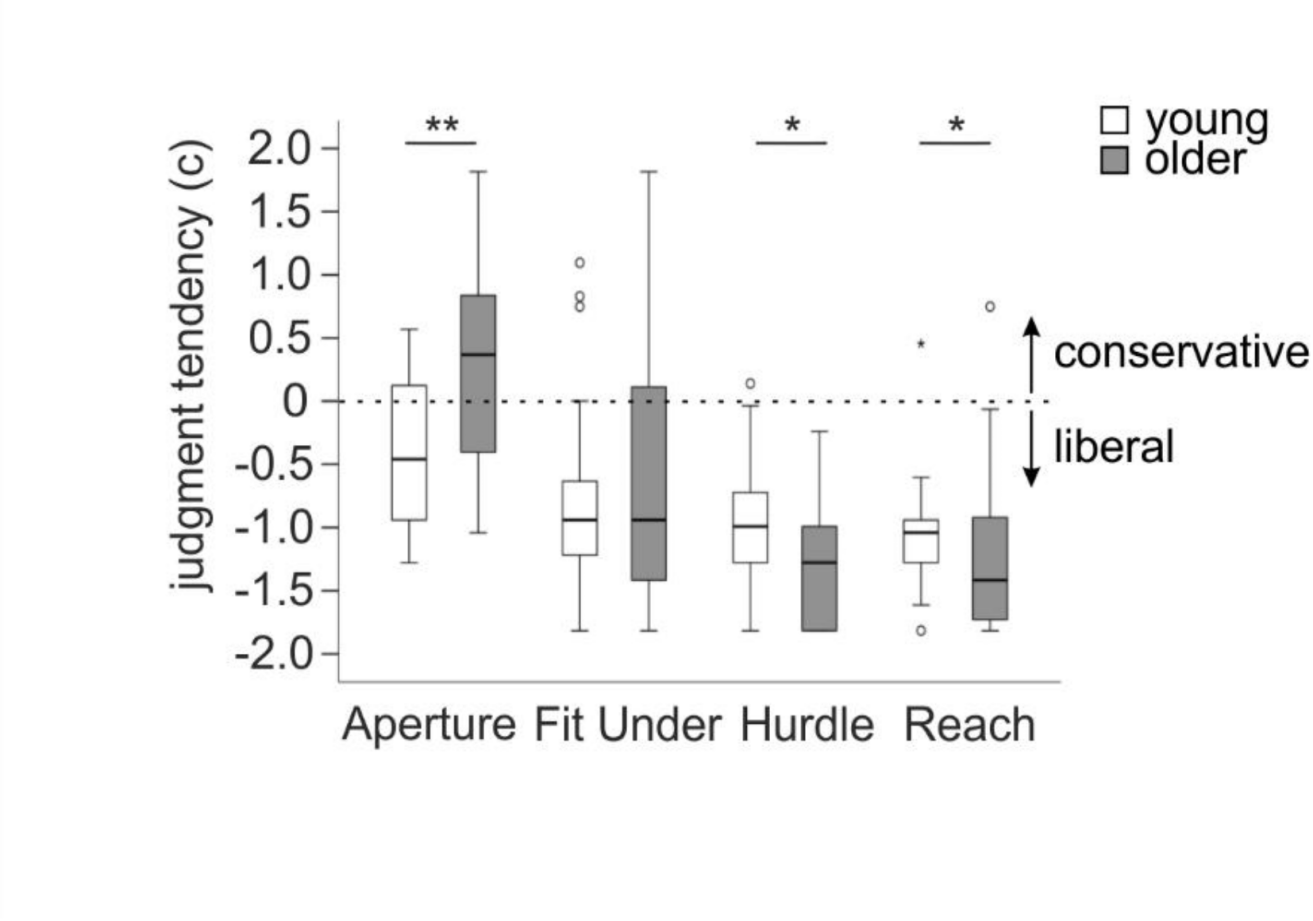
Boxplots for judgment tendency per task for the older subgroup (*n* = 24) and the younger control group (*n* = 24). Values closer to zero reflect a more ideal judgment tendency. Positive values stand for a rather conservative judgment tendency and negative values for a rather liberal judgment tendency. Note that older participants showed a more conservative judgment tendency in the Aperture Task and a more liberal judgment tendency in the Hurdle and Reach Task compared to the younger sample. *Note.* * *p* < .05, ** *p* < .01.

In the Aperture Task, the subsample of older participants (*n* = 24) showed a significantly more conservative judgment tendency than the younger participants (*n* = 24). In the Fit Under Task, there was no significant difference in judgment tendency between samples. In the Hurdle Task and Reach Task, older participants showed a significantly more liberal judgment tendency (see Fig. 3 for boxplots, Table 4 for descriptive data and Table 5 for Mann-Whitney *U*-test results).

**Table 4:** Descriptive data for the older subgroup (*n* = 24) and the younger control group (*n* = 24).

Variable	Aperture		Fit Under		Hurdle		Reach	
	*M*_dn _*[IQR]*		*M*_dn_* [IQR]*		*M*_dn_* [IQR]*		*M*_dn _*[IQR]*	
	Older *n* = 24	Younger *n* = 24	Older *n* = 24	Younger *n* = 24	Older *n* = 24	Younger *n* = 24	Older *n* = 24	Younger *n* = 24
acc	77.78 [65.74, 85.19]	85.19 [74.07, 88.89]	72.22 [60.19, 77.78]	70.37 [66.67, 77.78]	66.67 [55.56, 76.85]	75.93 [66.67, 80.56]	62.96 [55.56, 77.78]	72.22 [66.67, 77.78]
d’	1.83 [1.37, 2.30]	2.08 [1.68, 2.58]	1.48 [0.59, 1.88]	1.38 [1.06, 1.88]	1.21 [0.13, 1.83]	1.68 [1.21, 2.03]	0.93 [0.13, 1.83]	1.56 [1.21, 1.88]
c	0.37 [-0.43, 0.84]	-0.46 [-0.99, 0.20]	-0.94 [-1.42, 0.21]	-0.94 [-1.25, -0.62]	-1.28 [-1.82, -0.97]	-0.99 [-1.28, -0.66]	-1.42 [-1.77, -0.91]	-1.04 [-1.28, -0.94]

acc = accuracy, d’ = perceptual sensitivity, c = judgment tendency, *M*_*dn*_ = median,

*IQR* = interquartile range.

**Table 5:** Mann-Whitney *U*-test results for the comparison between the older (*n* = 24) and the younger (*n* = 24) sample per task.

Var.	Aperture				Fit Under				Hurdle				Reach			
	*U*	*p*	*1-β*	*r*	*U*	*p*	*1-β*	*r*	*U*	*p*	*1-β*	*r*	*U*	*p*	*1-β*	*r*
acc	207.50	0.095	0.373	0.24	281.00	0.890	0.052	0.02	183.00	0.029	0.586	0.31	191.00	0.044	0.515	0.29
d’	224.50	0.193	0.247	0.19	286.00	0.971	0.050	0.01	186.00	0.034	0.559	0.31	192.50	0.048	0.501	0.29
c	150.50	0.002	0.911	0.41	278.00	0.421	0.074	0.03	177.00	0.010	0.754	0.33	201.50	0.037	0.552	0.26

acc = accuracy, d’ = perceptual sensitivity, c = judgment tendency. Both *p*-values and Power (*1-β*) are reported two-tailed for acc and d’, and one-tailed for c. Power was calculated by using G*Power^49^.

#### Domain-specific risk-taking

Older participants showed significantly higher scores in risk-perception (*M*_*dn;*older_ = 5.92, *n* = 24; *M*_*dn;*younger_ = 5.00, *n* = 23; *U* = 163.00, *p* = .005) and lower scores in risk-taking (*M*_*dn;*older_ = 2.08, *n* = 24, *M*_*dn;*younger_ = 3.00; *U* = 132.50, *n* = 24, *p* < .001) than younger participants.

## Discussion

Adequate judgments of action opportunities are crucial to healthy aging; while physical and cognitive conditions change with increasing age, appropriate affordance judgments may be increasingly challenging. There is a lack of studies that systematically compare older people’s judgment tendencies in different types of tasks. In line with the literature covering judgment tendencies for single tasks^9^, with our within-subject design, we found systematically differing judgment tendencies in four different affordance judgment tasks depending on the type of task. Whereas participants tended to overestimate opportunities in affordance judgment tasks with distal boundaries like our Reach and Hurdle Tasks, action opportunities in tasks with proximal boundaries (e.g. Aperture and Fit Under Tasks) were more likely to be underestimated, or at least significantly less overestimated. When comparing older participants’ judgment behavior in these four tasks to a younger sample, we found more extreme judgment tendencies in the group of older participants. In the literature, several studies highlight the context dependency of affordances^54–56^, such as the influence of task constraints and overarching goals, which is important to the discussion on differences in judgment tendencies between tasks with proximal versus distal boundaries and will be addressed in more detail in the following. Here, we point out that age appears to pronounce these effects and we discuss potential influencing factors. In the following, we will first discuss our findings in the light of explanatory approaches derived from the literature that can either explain an overestimation or an underestimation in judgment tendencies specifically by older adults. Then, we will focus on explanatory approaches that may account for the task dependency of an over- versus underestimation in the same older adults.

Motives may be one influencing factor. In a study by Haines et al., one of the identified key factors influencing risk-taking behavior in hospitalized older adults was the participants’ desire to test their physical boundaries^57^. The authors highlight the importance of the internal motivation to test one’s physical boundaries as an important part of the rehabilitation process. The desire to push oneself to the limits may similarly apply to our healthy participants. As healthy aging goes along with declining physical capabilities, testing physical boundaries seems to be an important aspect of preserving independence^58^.

Beyond these motivational factors, the lack of an adequate adaptation of self-evaluation to weakened physical abilities may play a role. Examining older adults’ judgments on postural performance and stepping-over ability, Lafargue et al. argue that the updating process of internal models of action may be less efficient in older adults^59^, possibly due to cerebellar atrophy^60^ and shrinkage of the basal ganglia^61^. The authors suggest that decreased physical ability together with impaired updating of internal models may lead to an overestimation of action opportunities^59^. This consideration seems to fit older participants’ judgment tendencies in tasks with distal boundaries, as in the applied Hurdle Task and the Reach Task. Another possible influencing factor may be the individual’s perceived control over the task outcome. This is in line with the theory of planned behavior^62^ which highlights perceived behavioral control as a primary predictor of a person’s intention to perform an action^63,64^. Specific to tasks with distal boundaries, participants might have the notion of being more in control of the outcome. The perception of higher control of the outcome in turn could be linked to attributing action opportunities predominantly to one’s own bodily capabilities. The latter are more likely to be overestimated in older adults. As discussed above, this misjudgment could be due to motivational aspects and/ or an inadequate updating of declining physical capabilities^54,57,59^. However, this cannot serve as a general explanation, as there were significantly more conservative judgment tendencies for tasks with proximal boundaries. Instead, a differentiated view as a function of the type of task seems essential. Our diagnostic results on judgment tendency in the Aperture Task are in line with the results of previous studies showing rather an underestimation of action opportunities and applying this same task and analysis approach in other older samples^21,23^.

With growing age, older adults are confronted with a generally higher bodily vulnerability^1^. The more conservative affordance judgment behavior in the two tasks with proximal boundaries may reflect a more cautious behavior when the physical integrity is at risk. Tasks with proximal boundaries involve the potential risk of getting jammed and may evoke the awareness of a potential direct impact on the own body. Also, these tasks may lead to a restriction of freedom of movement, which is typically tried to be avoided. In line with this consideration, the study results by Comalli et al. suggest that people take the penalty of error into account in affordance judgments^24^. Older adults may perceive their physical vulnerability as being more in danger when solving tasks with proximal boundaries. Given these tasks’ constraints, it appears reasonable that perceived behavioral control^62,63^ may be lower. Tasks with proximal boundaries may include the notion of a more passive role with regard to the outcome. I.e., the risk of carrying out the task may be attributed rather to the setting than to participants’ own actions and, therefore, as being rather beyond their own control. Such a perceived limited personal influence may lead to more cautious judgments, i.e., an underestimation of action opportunities. A study by Horswill and McKenna showed that participants who imagined not being in control of a driving situation accepted a lower level of risk than participants who imagined being in control of the situation^65^.

This consideration is supported by our results on the applied control tasks, in which participants made gradual estimations on body-related size or related to objects. We showed that judgment tendencies in tasks with proximal boundaries (Aperture Task, Fit Under Task), were associated with older participants’ body-related size estimation, but not with general object-related estimation. The more participants overestimated their body-related size (hand width, body height), the more they underestimated their action opportunities. For the Hurdle and the Reach Task, there was no significant correlation between the evaluation of one’s own body size and overestimation of action opportunities in the actual tasks. This may imply that older adults rely more on assessing their own body measures particularly in tasks with proximal boundaries.

Even though we did find the expected disparity between the applied tasks with proximal versus distal boundaries, with both the Aperture and the Fit Under Task revealing a more conservative judgment tendency than the two tasks with distal boundaries, the Fit Under Task differed significantly in judgment tendency from the Aperture Task as well. In absolute terms, the Fit Under Task even revealed a rather liberal judgment tendency in our older participants. In other words, older participants underestimated their action opportunities in the Aperture Task, whereas they overestimated their action opportunities in the Fit Under, Hurdle and Reach Task. Other studies using fitting tasks, showed a rather conservative judgment tendency^23–25^, similar to our Aperture Task. However, these previous studies examined fitting tasks with horizontal boundaries, such as in the Aperture Task of the current study (e.g. walking through doorways, fitting the hand through an aperture), and not vertical fitting tasks, such as in the Fit Under Task. What might have led to this tendency towards overestimations in the Fit Under Task despite the tasks’ proximal boundaries? Beyond the direction of task-related boundaries, another aspect that might influence over- or underestimation of action opportunities in the applied tasks is varying perceived degrees of freedom when imagining executing the respective movement. In the field of motor control, the degrees of freedom problem has been known for a long time^66^. It states that “there are multiple ways for humans or animals to perform a movement to achieve the same goal”^67^ [p. 1]. Among other theories, Morasso describes the computational theory that highlights the cognitive aspect of the degrees freedom problem^67^. The core of the theory of what exactly and why this needs to be computed might be highly dependent on both the task-specific boundaries and the participant’s individual cognition. We expect the Aperture Task to imply the lowest number of perceived degrees of freedom, as the scope of action is strongly limited to the actual hand width. In the Fit Under task, participants were similarly very clearly instructed to imagine whether being able to stand upright and straight under the rod, limiting the action opportunities clearly to the participants’ body height. However, participants might have, more or less inadvertently, considered the possibility to, for example, slightly bend their knees or lower their head to still fit under the rod when the rod actually was too low. These potentially perceived degrees of freedom could have led to the unexpected results of a rather liberal judgment tendency in the Fit Under Task. Higher perceived degrees of freedom might in turn lower the perceived risk. The Fit Under Task revealed particularly high inter-subject variability in older participants, which could also be linked to different perceived degrees of freedom, as these may vary substantially between participants. This is again in line with the computational theory on the degrees of freedom problem that highlights the role of a cognitive agent^67^. Also younger participants may have been affected by perceived degrees of freedom. Due to different capabilities between the older and the younger sample in the Hurdle Task, different measurements had to be used (crotch height vs. maximal lifting height of the foot). This might have led to reduced perceived degrees of freedom in the Hurdle Task in the younger sample, which could have added to the less liberal judgments of the younger participants as compared to the older participants.

Potentially, the setting’s space may also play a role for judgment tendencies. A series of experiments looking at object-related affordances provide evidence that affordances rely not only on the mutual appropriateness of the features of an object and the abilities of an individual but also on the fact that those aspects fall within one’s own reachable space^68^. These studies argue that the action system for affordance perception is particularly relevant when the setting is within the person’s peripersonal space^68,69^. The aspect that the Fit Under Task was not within participants’ reach might have lowered risk perception (see Hunley and Lourenco^70^ for a review on the role of peripersonal space) and thereby might have led to a more liberal judgment tendency. It also cannot be excluded that potential age-related deficits in allocentric mapping contributed to the unexpected liberal judgment tendencies in the Fit Under Task. For example, in a study by Moffat & Resnick^71^, an analysis of map reproductions demonstrated that, in contrast to younger participants, older participants used proximal objects to locate a goal but disregarded information provided by more distal geometric room cues.

What factors might be associated with the extent of disparity of judgment tendencies between tasks? Our additional post-hoc analyses on body awareness revealed that the extent of disparity of judgment tendencies between tasks was associated with older participants’ body awareness. The higher their body awareness, the more extreme the disparity was. It is conceivable that a higher perceived awareness might be associated with a more conservative judgment tendency in tasks with proximal boundaries. In contrast, in tasks with distal boundaries, in which participants might perceive themselves to be more in control of mastering the task, a higher body awareness might be associated with higher action-related self-confidence, which could, in turn, lead to an overestimation of action opportunities.

Furthermore, our post-hoc analyses on cognitive variables revealed a significant correlation between the extent of disparity of judgment tendencies between tasks and alertness. The lower older participants’ alertness, the higher the disparity was. Participants with lower alertness might have relied more on general patterns or learned information (underestimation in the Aperture Task with proximal boundaries; overestimation in the Hurdle and Reach Task with distal boundaries), paying less attention to online information like the actual presented setting and its relation to their own body. In other words, the potential influencing factors mentioned above (i.e., motivational factors and the lack of adequate updating of actual capabilities leading to overestimation in the tasks with distal boundaries, respectively their vulnerability in tasks with proximal boundaries) may be more pronounced in older adults with lower alertness.

The comparison of the subsample of older participants to a younger control sample revealed the hypothesized results for the Aperture Task, the Hurdle Task and the Reach Task. In the Aperture Task, older participants showed a significantly more conservative judgment tendency than younger participants and showed rather an underestimation of action opportunities (for similar results see Finkel et al.^21,23)^. In the Hurdle and Reach Task, older participants showed a significantly more liberal judgment tendency as compared to the younger sample, reflecting a stronger overestimation of capabilities in the older participants (for similar results see e.g. Luyat et al.^10^ and Sakurai et al.^12^). Studying different types of tasks, our results show that older participants do not have a general tendency towards either an over- or an underestimation of capabilities, but they demonstrate more extreme judgment tendencies as compared to younger participants. The slight tendency in younger samples to overestimate their action opportunities in tasks with distal boundaries^9^ seems to become more pronounced in older adults. These results underline the relevance of the discussion above on the adaptation to changing bodily capabilities and related effects on the estimation of action opportunities in older adults.

With our current study results of the Aperture Task, we replicated those by Finkel et al. who showed that older participants decided in a more conservative way than a younger sample^23^. We also replicated their results on significantly higher scores in the DOSPERT scale in older participants as compared to a younger sample. However, besides higher risk-perception and lower willingness to take risk, our older sample mostly overestimated their action opportunities in the Fit Under, Hurdle and Reach Task. This leads to the assumption that higher risk-perception or lower willingness to take risks with regard to health and safety seem to play a subordinate role for judgments in the applied tasks. Instead, the mentioned motivational factors, body awareness and/ or inadequate updating on bodily capabilities seem to carry more weight.

With regard to limitations of the current study, it needs to be noted that our study investigated only static settings. Participants judged their action opportunities without being allowed to initiate the respective action. We cannot rule out that approaching, for example, the rod in the Hurdle or Fit Under Task might have changed the decision behavior. For example, a study by Hackney and Cinelli indicates that self-motion might affect perceived action capabilities^72^. The change in stability when walking might heighten the awareness of danger and could lead to deviating judgment tendencies. Therefore, from the current study, the assumptions about older adults’ affordance judgments are limited to static positions. All participants denied current movement restrictions. However, the measurement of actual capabilities can only refer to an instantaneous picture of participants’ abilities. In the current study’s design, we could not account for potential daily variations in participants’ range of motion. The study design does not allow any conclusion on the extent to which participants considered their specific form of the day in their judgments.

To our knowledge, this study is the first study investigating judgment tendencies in four different affordance judgment tasks with either proximal or distal boundaries in a within-subject design in an older sample. To examine more closely the considerations made as part of the discussion, task types should be further expanded and larger samples should be investigated in a cross-sectional design or even in longitudinal studies to underline the robustness of results on age effects and enrich the understanding of over- versus underestimation of action opportunities in affordance judgment tasks in older adults. A controlled investigation of potential modulating factors such as perceived risk when actually executing the applied tasks, perceived difficulty, perceived control, movement-related self-confidence, or perceived degrees of freedom in older participants could make a valuable contribution to the discussion.

Taken together, our findings shed light on potential factors influencing over- vs. underestimation of action opportunities in older adults in affordance judgment tasks. Contrasting task types seemed a useful approach for unraveling the mystery of older adults’ misjudgments of action opportunities. Our results support the hypothesis by Finkel et al. that the distinction of tasks presenting rather distal versus proximal boundaries can be an important modulating factor in judgment tendencies, particularly in older adults^9^. Based on our findings, we speculate that higher body awareness, lower alertness, and higher perceived outcome control lead to a stronger reliance on general patterns and (perhaps dated) learned information about our bodily capacities. Future research is needed to disentangle how much of the variance in judgment tendencies between tasks can be attributed to task-related boundaries (proximal vs. distal) or other modulating factors such as degrees of freedom, alertness, or body awareness. A deeper understanding of potential modulating factors for over- or underestimation of action opportunities in older adults could hold potential for developing tailored training opportunities in affordance judgments.

## Electronic supplementary material

Below is the link to the electronic supplementary material.

Supplementary Material 1

Supplementary Material 2

## Data Availability

Upon acceptance data will be provided within the manuscript or supplementary information files. For further inquiries please contact: Isabel.2.Bauer@uni-konstanz.de.
