# Supplementary material for: Older adults do not consistently overestimate their action opportunities across different settings
Source: Sci Rep. 2025 Feb 7;15:4559. doi: 10.1038/s41598-025-86790-6 (PMC11802724; doi:10.1038/s41598-025-86790-6)
Supplement: Supplementary file 1 — Supplementary Material 1 [file 41598_2025_86790_MOESM1_ESM.pdf]

Older adults do not consistently overestimate their action opportunities across different settings

Isabel Bauer[1, 2], Milena S. Gölz[1, 2], Lisa Finkel[3], Maddalena Blasizzo[1], Sarah E. M. Stoll[2, 4, 5] & Jennifer Randerath[1, 2, 5 *]

[1] Department of Psychology, University of Konstanz, Constance, Germany

[2] Lurija Institute for Rehabilitation Science and Health Research, Kliniken Schmieder, Allensbach, Germany

[3] Psychotherapy Training Center Bodensee (apb), Constance, Germany

[4] Department of Developmental and Educational Psychology, Faculty of Psychology, University of Vienna, Vienna, Austria

[5] Clinical Neuropsychology & Neuropsychological Psychotherapy, Institute of Psychology, University of Regensburg, Regensburg, Germany

* Corresponding author. Email address: J_Randerath@hotmail.com

Supplementary Information

	Aperture			Fit Under			Hurdle			Reach		
Variable	*Stat.*	*df*	*Sig.*	*Stat.*	*df*	*Sig.*	*Stat.*	*df*	*Sig.*	*Stat.*	*df*	*Sig.*
acc	.911	40	.004	.962	40	.200	.908	40	.003	.894	40	.001
d’	.939	40	.032	.954	40	.105	.909	40	.004	.929	40	.014
c	.965	40	.250	.926	40	.012	.907	40	.003	.865	40	< .001

Supplementary Table S1. Shapiro-Wilk Test results for older participants per task and variable.

Note. acc = accuracy, d’ = perceptual sensitivity, c = judgment tendency.

Variable		difference score Aperture-Hurdle		difference score Aperture-Reach	
	n	τ_b	*p*	τ_b	*p*
3D (spatial orientation; raw score correct)	40	-0.10	.400	-0.03	.788
Corsi (block-tapping test backwards; spatial span)	40	0.11	.363	0.00	.980
FGT (figural memory test; learning sum)	40	-0.03	.779	-0.01	.916
LAT (line orientation test; raw score correct)	40	-0.14	.239	-0.14	.225
NBV (N-back verbal; raw score correct)	40	-0.05	.679	0.14	.219
SIGNAL (signal detection; hits without late responses)	40	-0.12	.287	-0.06	.623
TOL (tower of London; raw score correct)	40	0.00	1.00	-0.00	.991
VISCO (visuoconstruction test; raw score visuoconstruction)	38	0.01	.910	0.02	.870
WAF Alertness (visual intrinsic alertness; time in ms)	40	0.38	<.001	0.24	.030
WIWO (vienna verbal fluency test; raw score)	39	-0.13	.359	-0.16	.149

Supplementary Table S2. Correlations (Kendall's tau, τ_b) between difference scores of judgment tendencies and neuropsychological test results for the older sample.
